# Study protocol for FAXAge: a randomized, controlled clinical trial of fasting and exercise to slow aging in humans

**DOI:** 10.1007/s11357-026-02362-0

**Published:** 2026-06-29

**Authors:** Emma Bundgård Fals, Emilie Caroline Springborg, Adam Bjørnholdt Berthelsen, Jonas Nyeman-Nielsen, Steen Larsen, Morten Scheibye-Knudsen

**Affiliations:** 1 Department of Biomedical Sciences, Faculty of Health and Medical Sciences, University of Copenhagen, Copenhagen, Denmark; 2 Center for Healthy Aging, Department of Cellular and Molecular Medicine, University of Copenhagen, Copenhagen, Denmark; 3 Clinical Research Centre, Medical University of Bialystok, Bialystok, Poland

**Keywords:** Aging Intervention, Older adults, Fasting, Exercise, Biological age

## Abstract

Biomarkers of aging, particularly DNA methylation-based clocks, have shown promise as tools to assess whether interventions may impact the rate of biological aging. Among possible interventions physical exercise has shown protective effects against many age-associated diseases, while time-restricted feeding (TRF) has shown metabolic benefits in preclinical models. The combined effect of exercise and TRF on aging biomarkers remains largely unexplored. In this 52-week four-armed, randomized, controlled trial (clinicaltrials.gov: NCT07207044) 240 healthy adults aged 65 and above will be allocated to four groups: combined cardio and strength training (EXE), TRF, combined EXE and TRF, or control. Participants will undergo assessments at baseline, 3, 6, and 12 months, with follow-ups at 2, 5, and 10 years. The primary outcome measure is Dunedin Pace of Aging DNA methylation age with secondary measures including RNA-sequencing, metabolomics, inflammatory markers, microbiome analysis, cognitive and physical measures. By deeply phenotyping participants, the Fasting And eXercise (FAXAge) study will provide novel insights into whether TRF, EXE, or a combination can slow or reverse biological aging in older adults.

## Background

Advances in medicine have among other factors led to an increase in global life expectancy across the twentieth and twenty-first century (Dattani 2023). In combination with declining birth rates, it is projected that by the year 2050, one in every six people worldwide will be above the age of 65 years; in Europe, this number is already one in four people (United Nations 2019). Although increasing life expectancy is positive, aging represents the largest risk factor for developing chronic diseases, and it has been suggested that aging per se should be regarded as a disease (The Lancet Diabetes Endocrinology 2018). Indeed, aging interventions could impact not only lifespan, but also the multitude of diseases associated with old age e.g., type 2 diabetes, cardiovascular disease, and cancer (Barzilai et al. 2016). An approach to impact all these chronic diseases could therefore be to treat aging itself thereby increasing both health- and lifespan.

Impacts of aging interventions in humans can be difficult to assess due to the long lifespan of humans. Efforts to find biomarkers of human aging that can be used to measure effects of human trials have therefore been explored for decades. About a decade ago, it was discovered that human aging tracks closely with changes in DNA methylation and subsequent work on these so-called aging clocks has shown that they are malleable for interventions (Moqri et al. 2023; Justice and Kritchevsky 2020). These biomarkers typically utilize machine learning methods to explore changes in multiple DNA methylation sites that appear to more strongly capture the complex changes in human aging compared to a single biomarker (Horvath and Raj 2018). Importantly, this approach of using machine learning on more complex datasets from physiological to biochemical biomarkers has proven efficacious in capturing the often subtle changes that occur with age, for instance nuclear morphology can be used to predict cell senescence (Teklu et al. 2025), while biological age can be determined by DNA methylation-based aging clocks (Horvath and Raj 2018) as well as portrait photos (Teklu et al. 2025). Based on previous work, we will utilize the Dunedin Pace of Aging DNA methylation clock that has been published to be responsive to cognitive and health-related outcomes (Belsky et al. 2020). Still no consensus has been achieved on the composition of biomarkers for the best model for biological age and little is known about how the different biomarkers relate to each other and to physiological outcomes (Moqri et al. 2023). Prospective intervention studies are therefore needed to establish and validate robust measures of biological age to build on the recent advances in the field (Moqri et al. 2024).

Being physically active is associated with increased life expectancy, potentially by attenuating age-related physiological alterations (American College of Sports Medicine 2009). These include the preservation of a healthier body composition with reduced total and abdominal fat, greater skeletal muscle mass, and higher bone mineral density, as well as improved cardiovascular function, enhanced insulin sensitivity, and lower levels of systemic inflammatory biomarkers (American College of Sports Medicine 2009). Collectively these adaptations contribute to the protective effects of exercise against most chronic age-related diseases (Pedersen and Saltin 2015).

Intermittent fasting and caloric restriction have been shown to increase lifespan in organisms from mice to rhesus monkeys (Willcox et al. 2007; Colman et al. 2009; Francesco et al. 2018) and improve health parameters in humans (Francesco et al. 2018). TRF is a type of intermittent fasting in which no calories are consumed for a period, typically 12–18 h per day. It is currently unknown whether this type of sustainable fasting improves markers of aging in older adults, although data suggests that periods of fasting can be healthy (Francesco et al. 2018). For instance, in rodents, TRF has shown protective effects against metabolic dysfunctions induced by the Western diet such as obesity, cardiovascular disease, hypertension and diabetes (Francesco et al. 2018). These benefits include reduction in body weight, increase in energy expenditure, improved glycemic control, and lower insulin levels, as well as decreases in hepatic fat and hyperglycemia (Francesco et al. 2018). The metabolic protection after TRF has been proposed to be due to metabolic adaptations, characterized by increased amounts of circulating ketones, while fatty acids, glucose and insulin remain at low levels (Francesco et al. 2018). Importantly, TRF has been shown to confer the major lifespan benefit of a calorie-restricted diet in mice (Acosta-Rodríguez et al. 2022). At the cellular level, it is believed that some of the effects of fasting are mediated through the activation of AMP-activated protein kinase (AMPK), recycling of cellular components via autophagy, reduced mTOR activation and increased mitochondrial biogenesis (Francesco et al. 2018). However, loss of muscle and bone mass may limit the use of this intervention in older adults where this could be a serious side effect (Fernández-Rodríguez et al. 2024). Fasting combined with exercise could be a means to mitigate the possible catabolic effect of intermittent fasting (Laurens et al. 2021).

**Fig. 1:**
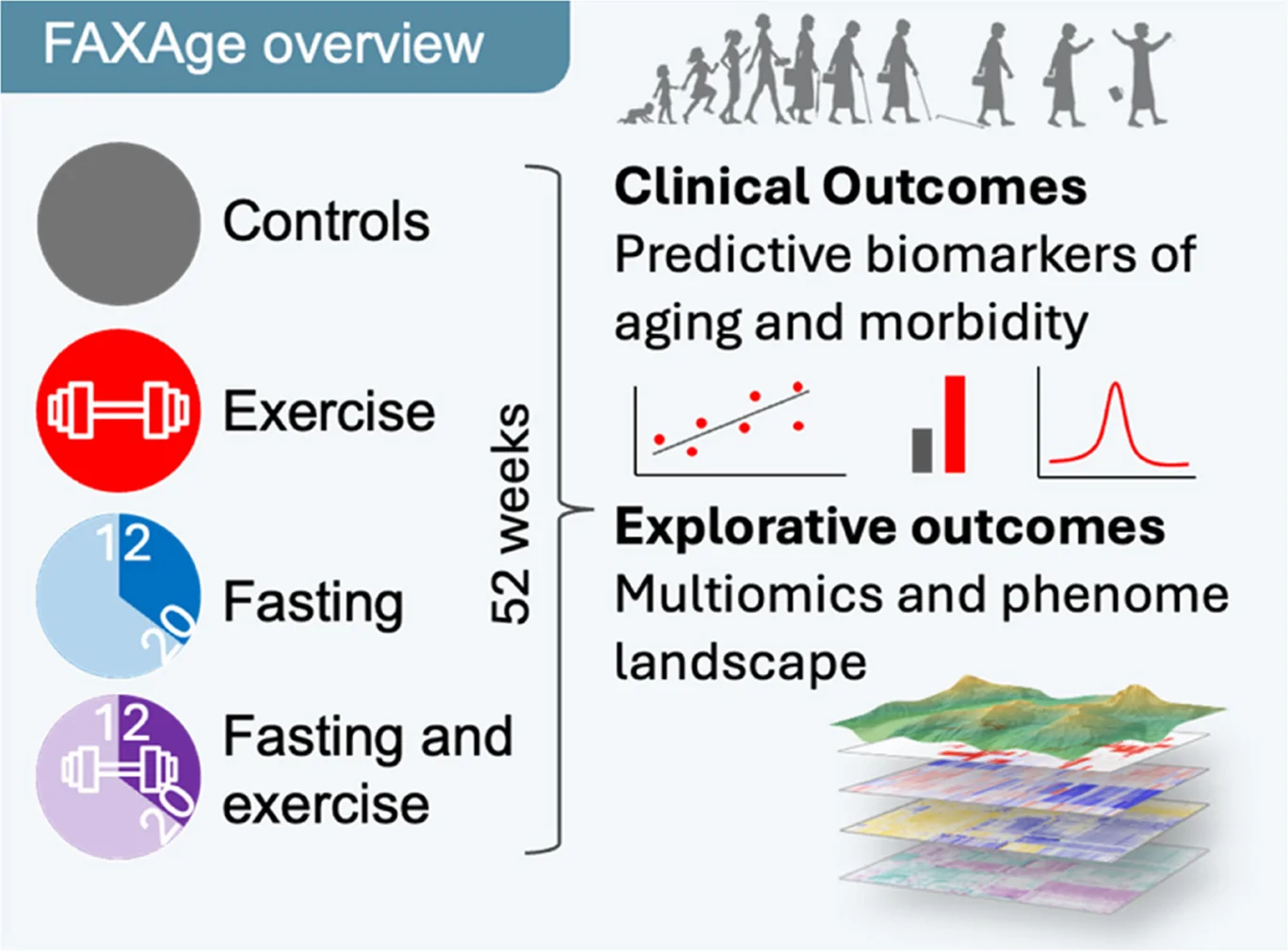
Overview of the study

To address these issues, this one-year case–control clinical trial will investigate biological age, as a primary outcome measure using DNA methylation, and secondary outcome measures including physiological and molecular outcomes. Importantly, the study will have a 10-year follow-up period potentially allowing the potential identification of long-term health outcomes. This may allow us to answer the following key questions which are critically needed for the field: 1) Which markers of biological age are clinically relevant? 2) Is biological age amenable for interventions? 3) Which biological age measurement, DNA methylation, omics-based, digital, hematological, physiological or others, is most reproducible? 4) Is biological age predictive of health and future outcomes? 5) Are the non-invasive biomarkers as good as the more invasive biomarkers? 6) How do different biomarkers associate with each other and other health parameters? To answer these questions, a 52 weeks four-armed randomized, controlled intervention trial will be conducted with subjects randomized to either a combined cardio and strength exercise (EXE), fasting via time-restricted feeding (TRF), combined TRF and EXE (FAX), or control group (Fig. 1). This trial will provide new insight into whether TRF and exercise may reduce or potentially reverse the rate of human aging.

We hypothesize that exercise, intermittent fasting and a combination of the two will provide similar, superior benefits to DNA methylation epigenetic age measured with Dunedin Pace of Aging clock (Belsky et al. 2020) when compared to controls after 52 weeks. It is furthermore hypothesized that the interventions will provide similar, superior benefits to CRP, TNF-α, IL-6, IL-8, NAD +, hematologic age, cell senescence in blood smears, cognitive function, transcriptional age (RNA-sequencing), metabolomic changes, microbiome changes, functional age (handgrip strength, gait speed, VO_2_max), body composition, vocal age, and photo age when compared to controls after 52 weeks.

## Methods

### Study design

**Fig. 2:**
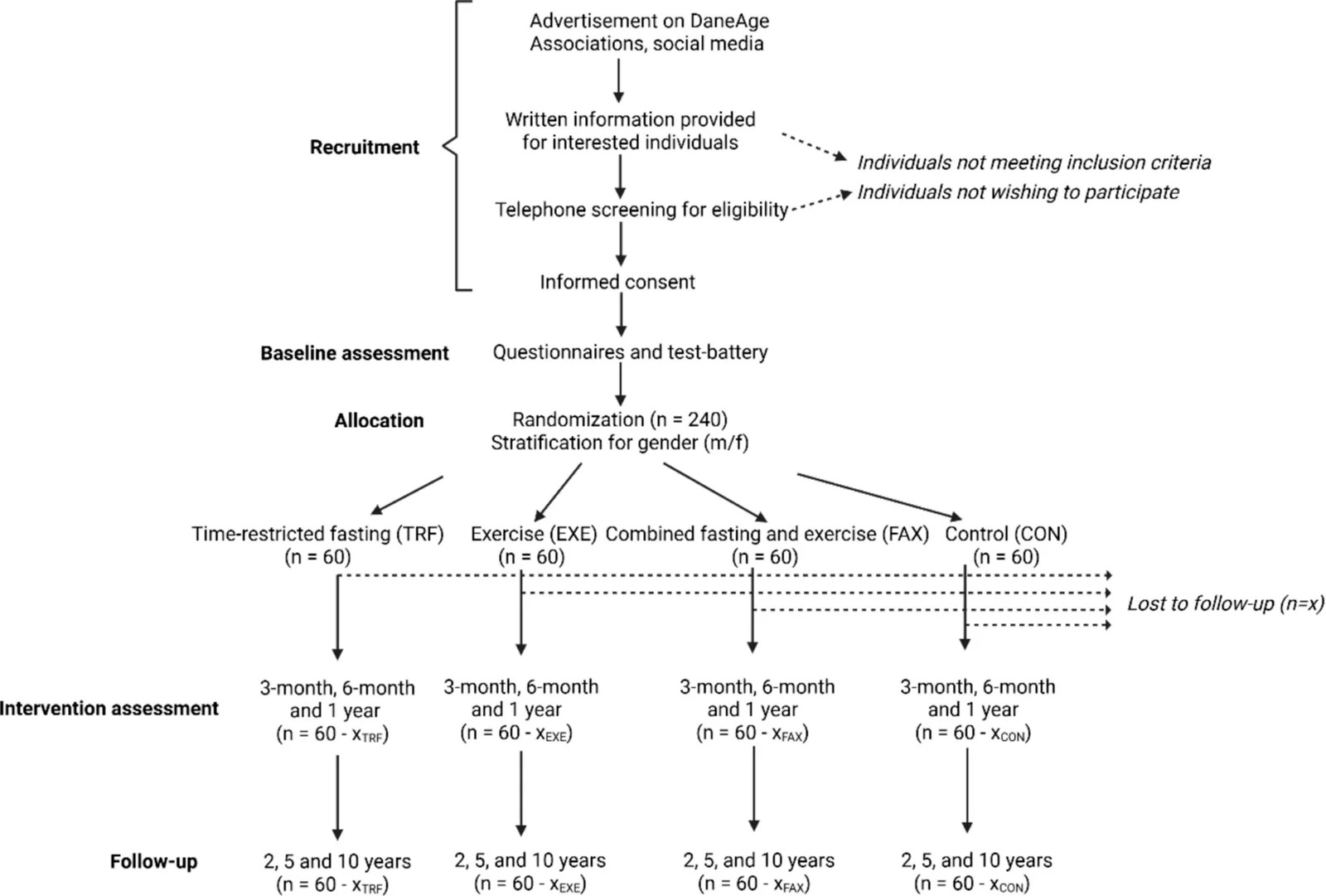
Flowchart of the FAXAge experimental set-up

The study visits will be conducted at the Department of Biomedical Sciences, University of Copenhagen. 240 healthy community-dwelling elderly aged 65 and older in good general health will be recruited and block randomized to either of the interventions for 52 weeks. Assessments are carried out at baseline, 3 months, 6 months, and 12 months and followed up after 2 years, 5 years and 10 years (Fig. 2).

### Participants, recruitment strategy, and randomization

Participants are excluded if they have received another investigational drug or intervention within 1 year, as prior treatments could influence the responses observed in this study, making it difficult to isolate effects to the current intervention. Individuals engaging in more than one hour of systematic strenuous exercise or strength training a week are excluded to maintain similar baseline activity levels across participants and reduce variability in fitness, making the effects of the intervention clearer. Individuals already practicing time-restricted eating are excluded, as they will not show clear pre- and post-intervention responses. We wish to include a representative part of the healthy elderly population; however, we expect significant bias in our data if we only include individuals without chronic diseases. For this reason individuals with severe or dysregulated diseases are excluded, as the disease state may independently impact the intervention outcomes, while allowing one well-managed chronic disease helps limit heterogeneity and risks for the participants. Smoking is an exclusion criterion due to its effects on cardiometabolic health, inflammation, and exercise capacity, all of which could confound study results. Finally, individuals using systemic glucocorticoids, androgens, or antiandrogens are excluded because these medications have strong effects on metabolism, muscle adaptation, appetite, and glucose tolerance, potentially masking or exaggerating the effects of time-restricted feeding and exercise interventions. Participants are recruited via online advertising, on social media, and in the DaneAge Association membership magazine. Participants will be randomized according to a permuted block randomization scheme with a block size of 4, stratified according to biological sex. The randomization list will be generated prior to commencing the study.

### Interventions

Participants randomized to the EXE and FAX group will perform supervised resistance training two times per week as well as cardio training at home two times per week monitored by fitness watches to track heart rate. This combined approach is chosen since both high muscle strength and cardiorespiratory fitness are independently associated with lower all-cause mortality, whereas low levels correlate with increased risk (Schnohr et al. 2025). Accordingly, this intervention is designed to enhance both muscle strength and cardiorespiratory fitness, enabling the investigation of their association with changes in markers of biological age.

The strength training intensity will be individualized and increased over time according to changes in the participant’s training status, aiming to maintain a similar relative load across participants by adjusting loads to a target range of repetitions in reserve. The training program will consist of the same exercises over the 52 weeks and is designed in a progressive manner as this approach has been shown to enable long-term adaptations in muscular strength through progressive increases in mechanical loading and total training stimulus, thereby limiting training plateaus over time (American College of Sports Medicine 2009). It will consist of approximately 1 h of strength training per session with at least 48 h between each session. Participants will perform a standardized, machine-based (Technogym S.p.A, Cesena, Italy) strength training program involving exercises for the lower and upper body. The program includes both multi-joint (leg press, chest press, lateral pulldown and low row) and single-joint (leg extension and lying leg curl) exercises, with an emphasis on multi-joint movements, which have been shown to elicit greater improvements in muscle strength and contribute to improvements in cardiorespiratory fitness compared with single-joint exercises when total training load is matched (Paoli 2017). This effect has been attributed to a higher oxygen demand associated with the involvement of a larger active muscle mass (Paoli 2017).

The training protocol is based on the LISA study and guidelines to resistance training of older adults from the American College of Sports Medicine (American College of Sports Medicine 2009; Eriksen et al. 2016). The program is initiated with a 4-week familiarization phase characterized by high-repetition, low-load training, to ensure proper technique, allow musculoskeletal tissue adaptation and to reduce the risk of injury (Signorile 2013). The training period consists of 4 blocks of 13 weeks (the first block includes the 4-week familiarization phase). Each block begins with three sets of 12 repetitions per exercise, with the number of repetitions decreasing in a stepwise manner every three weeks, ultimately concluding with four sets of six repetitions in the last three weeks. Within each block, training will be organized into consecutive 3-week phases during which repetition range, target repetitions in reserve, and number of sets will be maintained constant, allowing sufficient time for adaptation before progressing to the next phase. Load is continuously adapted for each training session based on participant-reported repetitions in reserve in the previous session. When the upper target is reached and maintained for ≥ 2 sessions, load is increased. Target repetitions in reserve vary for each 3-week phase in accordance with the prescribed number of repetitions – the fewer repetitions, the fewer target repetitions in reserve. Every 13th week a deload phase is implemented to facilitate recovery and mitigate fatigue development (Bell et al. 2023). During this week, training volume is reduced ~ 30 to 50% by reducing the number of sets for each exercise and increasing target repetitions in reserve while maintaining the number of repetitions. At the end of each block, an indirect one repetition maximum load test is performed using the Brzycki formula (Brzycki 1993) at a target of six repetitions, to test alignment between participant-reported repetitions in reserve and one repetition maximum based loading to ensure load is progressive (American College of Sports Medicine 2009). After completion of each block, the same progression pattern is repeated in the subsequent block with absolute loads adjusted from the one maximum load test, starting at ~ 65% of calculated one repetition maximum load. Training sessions will be supervised and will take place at the Department of Biomedical Sciences, University of Copenhagen.

Cardio training will be of self-chosen modality and at moderate to vigorous intensity (> 70% HRmax) since vigorous-intensity exercise may result in greater increases in aerobic capacity than moderate-intensity (Gormley et al. 2008) and that participation in vigorous activities is associated with lower mortality rates (Lee and Paffenbarger 2000). The desired duration will increase from 30 min per session in the first month, 45 min per session in the second month to 60 min per session in the following 3–12 months. Adherence, workout duration, and average and maximal heart rate will be monitored by fitness watches provided to each participant.

Participants randomized to the TRF group will be instructed to abstain from any caloric intake during the targeted fasting window of 16 continuous hours and consume ad libitum during the 8-hour eating window. Participants can choose their preferred eating window and are encouraged to drink plenty of water during fasting. Participants will receive an adherence diary including an eating time log for noting the time of first and last calorie consumption each day.

Participants randomized to the FAX group will be instructed to both exercise and fast during the 52-week intervention.

### Measurements

**Fig. 3:**
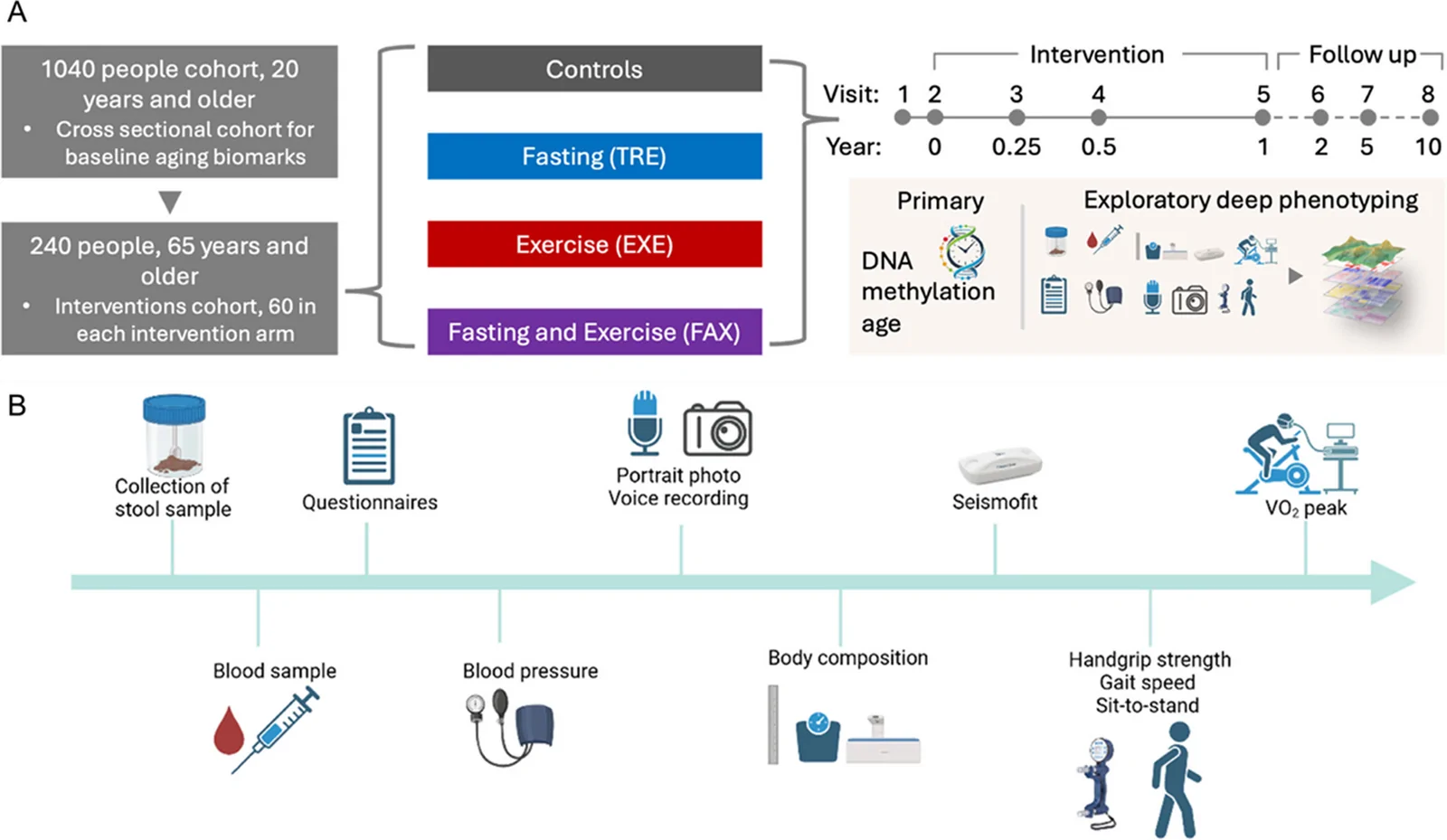
Visualization of the test-battery. **A**. Participants are tested at baseline, after 3 months, 6 months and 1 year, and follow-up will be performed 2, 5 and 10 years after baseline. **B**. A stool sample is collected at home for microbiome analysis within 18 hours of the test day and brough to the lab. A blood sample is collected to assess multiple blood markers after which participants fill out questionnaires, have their blood pressure measured and a digital photo is taken and a voice recording ise performed. Body composition is measured with a DXA scan and VO_2_max is estimated with Seismofit. Lastly participants perform a physical test battery consisting of handgrip strength, gait speed and sit-to-stand tests as well as a graded cardiopulmonary VO_2_peak test

Participants go through a battery of tests before the intervention (Fig. 3), 3 months and 6 months into the intervention and after the intervention. Additionally, follow-up testing is performed at 2, 5 and 10 years after the intervention. Follow-up testing is deemed critical because it may allow us to discover long-term beneficial or detrimental effects of these interventions. In the section below we will go through the different data modalities that will be gathered as part of the intervention and the rationale for why these measures were chosen. Tests are performed according to defined standard operating procedures (SOPs) in the same order for all participants at all visits, and to ensure a minimum of bias, test leaders are instructed to not look at the participants’ previous test results before a new test. In addition, biological samples will be processed using harmonized workflows with batch tracking, internal controls, and standardized normalization procedures where applicable. For molecular analyses, raw data and metadata will be retained to allow reprocessing and harmonized downstream analyses as analytical methods evolve. For phenotypic and physiological measurements, personnel will be trained in predefined acquisition procedures to minimize interoperator bias.

#### Primary outcome measure

As the primary outcome measure of the trial, Dunedin Pace of Aging DNA methylation analyses (Belsky et al. 2020) will be performed using the Illumina 450 K array on peripheral blood mononucleated cells. It was chosen to isolate these cells instead of performing investigations on whole blood to reduce the potential effect of blood composition changes that may be a side effect of interventions. Raw DNA methylation data will be stored to enable re-analysis using both current and future generations of epigenetic clocks and computational pipelines. Long-term follow-up samples will initially be analyzed using the same methylation platform and primary clock algorithms to ensure longitudinal consistency, while harmonized re-analysis with updated methods may subsequently be performed as the field evolves. In addition, specific DNA methylation changes in the epigenome will be investigated in secondary explorative outcomes in connection with RNA-sequencing results with appropriate false discovery rate corrections made.

#### Exploratory outcome measures

Blood work

Venous blood is collected following a 12-h overnight fast at the start of the test day and used for analysis of multiple parameters (Table 1).

**Table 1:** Measured blood markers

Category	Blood marker
Inflammatory markers	TNF-α, IL-6, IL-8, CRP
Glycemic control	HbA1C, Glucose
Hematology	Hb, Erythrocytes, Hct, MCH, MCHC, MCV, Platelets, RBC, Leukocytes, Basophils, Eosinophils, Monocytes, Lymphocytes, Neutrophils
Molecular tests	DNA methylation, RNA-sequencing, Metabolomics
Biochemical	ALAT, ASAT, Alkaline phosphatase, Albumin, Urea, Total protein, Iron, Bilirubin, Lactate acid dehydrogenase, Calcium, Creatinine, Total cholesterol, HDL, LDL, Triglycerides, Selenium, Q10, NAD +, Sodium, Potassium, Chloride
Computational tests	Senescence prediction on blood smears (Teklu et al. 2025; Heckenbach et al. 2022)

Measurement of inflammatory markers is important, as chronic, low-grade inflammation is a hallmark of aging and has been linked to an increased risk of frailty and mortality (Flanagan et al. 2020; Arosio et al. 2023; Ferrucci and Fabbri 2018). Glycemic control markers are measured because impaired glucose regulation is common among older adults (Dubowitz et al. 2014) and is associated with elevated risk of most chronic age-associated diseases (Schnell and Standl 2006). Complete blood cell count parameters are assessed since aging influences hematopoiesis, and white blood cell counts serve as predictors of all-cause mortality (Groarke and Young 2019).

RNA-sequencing and DNA methylation changes in blood mononucleated cells will provide insights into transcriptional and signaling processes at the cellular level. Metabolomic profiling will be performed on plasma to assess the changes, exercise and fasting induce at the molecular level and what adaptations and cellular pathways are involved. Lastly, biochemical and metabolic markers are measured to detect organ system decline and age-related metabolic disturbances. Combined with physiological measures this will provide a comprehensive understanding of how fasting and exercise influence aging.

A finger prick is also performed on the tip of the middle finger for thin blood smear senescence prediction as recently described (Heckenbach et al. 2022).

Stool sample

Changes in the gut microbiome have been seen with age and may predict health outcomes in the elderly (Bradley and Haran 2024; N V 2025). Notably, gut microbiome is strongly affected by feeding habits and we expect TRF will have a considerable effect on this (Paukkonen et al. 2024). For that reason, all participants will be given a feces kit to collect a stool sample prior to each test day. The stool sample will be collected on the test day and stored at −80 degrees Celsius until analysis. For analysis, we will perform 16S rRNA sequencing.

#### Questionnaires

To gain understanding of overall health, sleep quality, and cognitive decline participants answer the following questionnaires:

Health examination

Participants will answer a modified version of the Danish Health Examination Survey questionnaire, including questions about chronic diseases, dietary habits, alcohol, stress, physical activity and for female participants their menstrual cycle (see appendix).

Sleep quality

Pittsburgh Sleep Quality Index (PSQI) questionnaire will be used to assess subjective sleep quality and disturbances over the past month. Questions include assessment of duration, latency, efficiency, disturbances, and daytime dysfunction. Sleep will be further evaluated through wearables.

Montreal Cognitive Assessment (MoCA)

The MoCA Full test is performed to assess a wide range of cognitive domains, including memory, attention, executive function, language, visuospatial abilities and orientation, to possibly identify mild cognitive impairment (MCI). This allows for identification of subtle cognitive deficits and is better suited in healthy elderly than the mini-mental state examination (Nasreddine et al. 2005). Nevertheless, several considerations should be made concerning the MoCA test as a secondary outcome. First, the MoCA test may be subject to ceiling effects due to healthy individuals scoring close to max (Bernier et al. 2023), second it is possible that a one-year trial will not yield sufficient changes in MoCA scoring during the trial period (Bernier et al. 2023), third repeat testing may impact the outcome of the test. Nevertheless, we chose this test due to the feasibility in terms of time-management during trial days, the clinical application of test and because we have a significant long-term follow-up time where cognitive decline is expected and where the interventions may impact these outcomes.

#### Vitals

Participants’ resting pulse and blood pressure are measured three times on the left arm using an electric blood pressure monitor in a seated position with both feet flat on the floor and the arm resting on the table. Blood pressure is measured after filling out questionnaires to ensure that the participant is relaxed. Three consecutive readings are obtained automatically, and the mean of the values is used. If the variability between the first three consecutive readings exceeds threshold, the blood pressure measurement is repeated and the outlier is discarded (University of Maryland 2009).

#### Portrait photo and voice recording

A portrait photo as well as a voice recording, answering a standardized question, will be obtained of each participant. For facial photographs, standardized image acquisition procedures will be implemented, including controlled lighting, fixed camera positioning and distance, neutral facial expression, and standardized background conditions. Participants will be instructed to avoid excessive makeup, major facial accessories, and recent cosmetic procedures when possible prior to imaging sessions. Audio clips are expected to be 1 min. Both will be used for determination of the participants’ biological age by using facial image-based and voice-based age prediction algorithms using deep neural networks trained to predict age (Teklu et al. 2025; Kwasny and Hemmerling 2021).

#### Body composition

Body composition and bone mineral density are evaluated with dual energy X-ray absorptiometry (DEXA)-scan (Lunar iDEXA, Madison, Wisconsin, USA) using enCORE software, V.18 (Krugh 2025). A whole-body scan is performed to determine fat-percentage, visceral fat mass, lean body mass and bone mineral density.

#### Handgrip strength

Grip strength is measured to assess the maximal isometric handgrip strength in kilograms with a Jamar Smart Hand Dynamometer (Jamar, Nottinghamshire, UK). Participants are seated in a chair with a straight back with the elbow bent in a 90-degree angle. The dynamometer is pointing vertically upward. The measurement is a 5 s maximum-effort measure and is repeated three times on each hand with one minute rest in between trials on the same hand. The participants are verbally encouraged during each maximum-effort measure. The highest value obtained for each arm is reported and used for data analysis.

#### Gait speed

Participants’ gait speed and function are assessed by a standardized 4-m test, where participants are instructed to walk two times—once at their normal pace and once as fast as they can. Participants are also video recorded while walking and gait speed, acceleration, balance, cadence, step length, posture and joint angles will be estimated using Tracked Biotechnologies’ TrackedGait system (Tracked Biotechnologies, Virginia, USA).

#### Sit-to-stand

A 30 second sit-to-stand test is performed to assess strength in the lower extremities. The test is performed using a chair (45 cm) without armrest. Participants are shown how to perform the test and instructed to place their feet flat on the ground, cross the arms and hold them against the chest. Participants must stand fully and contact the seat fully. The test is completed two times and the score is the highest number of full stands completed within 30 s.

#### ***VO***_***2***_*** measurements***

Estimated VO_2_max

VO_2_max will be estimated at rest using seismocardiographic measurements (Ventriject, Copenhagen, Denmark) (Hansen et al. 2023). This will be done immediately after the DXA scan where participants have been lying down relaxed for ~8-10 minutes.

Direct measure

A cardiopulmonary exercise test (CPET) is performed on a Monark LC7TT bike (Monark, Varberg, Sweden) using the Quark CPET metabolic cart (Cosmed, Rome, Italy) to determine the peak oxygen consumption rate using indirect calorimetry. Participants perform a graded exercise test with warm-up and increment intensity adjusted to their Seismofit-estimated VO_2_max. Estimated peak power output is calculated from the estimated VO_2_max, a sex-specific coefficient, body weight and a regression-based constant interpreted from Eriksen et al (2014). From estimated peak power output, warm-up intensity and increment size are calculated to reach a total test duration of 10–12 min including a 5-min warm up (Buchfuhrer et al. 1983).

#### Activity monitors

Activity monitors (Huawei Band 10, Huawei, Shenzhen, China) will be provided to all participants to monitor daily activity. The activity monitors will be used by EXE and FAX participants to monitor home workouts of self-chosen modality and are used to ensure that average heart rate is > 70% HRmax for each session. For CON and TRF the activity monitors are used to assess average activity level. Furthermore, the monitors are used to assess sleep quality, which will be compared to the PSQI scores.

## Retention

### Biweekly phone calls

To increase participant retention and compliance with the randomly prescribed interventions, personalized attention will be provided through biweekly phone calls throughout the intervention for all four groups. This will give an opportunity to discuss individual challenges and tailor solutions to help participants adhere to the various interventions. However, there will not be applied pressure on individuals to continue their enrollment if they wish to withdraw from the trial.

### Strategy for follow-up assessment

At the end of the study participants will be encouraged to continue with the respective protocols which may allow us to determine differences in health outcomes between individuals that are continuing these behavioral outcomes and those that do not. Participants will be reminded by email ~ 2 months prior to follow-up assessments. The email will be followed up by a phone call shortly after to schedule a date for the follow-up test day. An email with confirmation of the appointment will be sent right after the phone call. A reminder email confirming the agreed time and date will be sent out ~ 2 weeks prior to the follow-up as well as a reminder email with practical information the day before the test day.

### Statistical considerations

Sample size determination for the interventions trial is conducted using the primary outcome DNA methylation measured at 52 weeks for the pooled three intervention groups (EXE, TRF, FAX) versus the control group (Table 2). Biological age prediction using epigenetics yields a standard deviation of ~10% (Guan et al. 2024). Assuming an expected pooled effect size for the intervention groups versus the control group is considerable (Cohen’s d = 1.0). With a probability of a Type I and Type II error set at α = 0.05 and β = 0.20, respectively, the estimated sample size was 40 participants allocated in a 1:3 ratio (controls:intervention groups). Our 3-month pilot study (Slowage, clinical trials id: NCT05593939) as well as a similar previous 1-year training intervention study with older individuals showed low dropout rates around 5–10% (Gylling et al. 2020). To accommodate the possibility of a higher dropout rate, a total of 240 participants will be recruited whereby 60 participants will be included in each of the four groups (EXE, TRF, FAX, CON), allowing for a 33% dropout rate. Data from dropouts will not be included in the primary outcomes but may be used in exploratory outcomes. Statistical analysis of outcomes will be done blinded by the outcome assessor. For omics-derived statistics, such as DNA methylation and transcriptomics, results will be corrected for multiple comparisons using false discovery rate control, for example Benjamini–Hochberg FDR at 5%.

**Table 2:** List of outcomes

Procedure	Baseline	3 months	6 months	1 year	Follow-up (2, 5, 10 years)
DNA methylation (primary)	X	X	X	X	X
Questionnaire	X	X	X	X	X
MoCA	X	X	X	X	X
Height	X	X	X	X	X
Weight	X	X	X	X	X
Body composition	X	X	X	X	X
Blood pressure	X	X	X	X	X
Blood samples	X	X	X	X	X
Microbiome sequencing	X	X	X	X	X
Handgrip strength	X	X	X	X	X
Gait speed	X	X	X	X	X
Seismofit	X	X	X	X	X
VO_2_peak	X	X	X	X	X
Voice recording	X	X	X	X	X
Portrait photo	X	X	X	X	X

It should be noted that, although DNA methylation clocks are among the most established biomarkers of biological aging, they remain surrogate measures that may be influenced by technical variation, tissue source, immune-cell composition, and algorithm selection.

### Data integration

To assess the biological relevance of omics changes, data such as DNA methylation will be integrated with RNA-sequencing, metabolomics, inflammatory and hematological markers, microbiome profiling, and physiological outcomes including VO₂peak, body composition, muscle strength, gait speed, cognition, and blood pressure, while exploratory CpG- and pathway-level analyses will be compared with transcriptomic gene set enrichment and phenotypic changes to evaluate biological coherence.

### Personal information

The identities of all participants will be known and stored at the laboratory site at the University of Copenhagen (UCPH). This information will not include the participants’ contact details and identification details. Instead, each participant and their associated research data will be identified by a unique study identification number. Data will be stored and managed in full compliance with regulatory bodies and according to GDPR. Study data will be managed on the secure, web-based software platform REDCap where it will be secured and password-protected and only accessible for research staff working on the project. At the conclusion of the study, all study databases will be de-identified and archived at UCPH.

## Discussion

The FAXAge study investigates the combined and individual effects of physical activity and TRF in a year-long randomized intervention trial. To our knowledge, no previous study has combined these two interventions in older adults, potentially providing novel insights into how dietary manipulation and structured exercise interact to influence the rate of aging. While the role of physical activity in promoting healthy aging is well supported by research and clinical evidence (Strasser and Burtscher 2018; Gylling et al. 2020; Imboden et al. 2019), little is known regarding the effects of exercise on rates of aging. Further, while work in preclinical models indicates that TRF is among the strongest influencers of aging and lifespan in animals; it is unknown if sustained intermittent fasting interventions can influence health and rates of aging in humans.

This trial will provide important evidence on the effects of intermittent fasting and exercise, both independently and in combination, on molecular biomarkers of biological aging in older adults. The use of multiple measures of biological aging, including molecular, physiological, and digital biomarkers, will enable a multidimensional assessment of aging processes. This integration of lifestyle interventions and biomarkers will address a knowledge gap regarding whether the rate of aging can be reduced or potentially slowed through lifestyle interventions, an important finding that could inform health policies for societies across the globe.

Should TRF, exercise, or the combination prove effective, these strategies could represent affordable, scalable, and safe strategies to promote healthy aging. This intervention study could potentially shape governmental lifestyle recommendations for healthy aging, as such interventions may reduce the prevalence of age-related diseases like cardiovascular disease, diabetes, and cognitive decline, thereby promoting independence among older adults. Enhanced physical function and metabolic health also reduce the frailty and fall risk, leading to fewer hospitalizations and lower care needs (Caicedo-Pareja et al. 2024; O’Hoski et al. 2020).

Beyond health benefits, these interventions have economic implications. Given they lower disease burden and subsequently the healthcare utilization, they reduce the financial burden on health systems and extend the productive lifespan of older adults. Research suggests that an increase in life expectancy by 1 year is worth 38 trillion US dollars, while an increase in life expectancy by 10 years is worth 367 trillion US dollars (Scott et al. 2021). Promoting fasting and exercise in elderly populations, therefore, holds potential to generate significant economic returns.

Additionally, improved physical and cognitive health supports greater social participation and community engagement among older adults, which in turn enhances quality of life (Fried 2016). Indeed, societal participation may not only increase perceived health but also reduce mortality, cognitive disability, frailty, and prevalence of depression (Fried 2016). Thus, giving older adults the guidelines for healthy aging, this intervention could empower them to contribute to society, benefitting both their quality of life, society and the economy. Furthermore, healthy aging may encourage older adults to reenter or remain in the workforce, potentially increasing annual GDP, thus benefitting the economy (Scott 2021).

A limitation of the present study is that the interventions are performed in relatively healthy older adults, in whom age-related molecular and physiological changes may occur more slowly and baseline biomarker variability may be lower than in diseased populations. Consequently, intervention-induced changes may be modest, particularly in cognitive measures of healthy individuals, and more difficult to detect over the 52-week intervention period despite the comprehensive longitudinal phenotyping performed in the study. Nevertheless, data suggest that muscle function can be significantly and lastingly impacted by strength training as used in this protocol (Gylling et al. 2020).

Follow-up assessments are performed after 2, 5 and 10 years which will also include questionnaires regarding behavior such as food and exercise. This will allow assessment of whether the potential beneficial effects of the interventions are sustained over time after the intervention ends. Furthermore, it will identify potential delayed or long-term benefits or adverse effects that might not be evident immediately after the intervention. Follow-ups will provide data on how fasting and exercise influence outcomes like disease risk and mortality in the elderly.

## Data Availability

Not applicable.
